# Dopamine Signaling in Substantia Nigra and Its Impact on Locomotor Function—Not a New Concept, but Neglected Reality

**DOI:** 10.3390/ijms25021131

**Published:** 2024-01-17

**Authors:** Michael F. Salvatore

**Affiliations:** Department of Pharmacology & Neuroscience, University of North Texas Health Science Center, Fort Worth, TX 76107, USA; michael.salvatore@unthsc.edu

**Keywords:** substantia nigra, dopamine, tyrosine hydroxylase, dopamine receptor, striatum, reuptake, phosphorylation, nigrostriatal, Parkinson’s disease, aging

## Abstract

The mechanistic influences of dopamine (DA) signaling and impact on motor function are nearly always interpreted from changes in nigrostriatal neuron terminals in striatum. This is a standard practice in studies of human Parkinson’s disease (PD) and aging and related animal models of PD and aging-related parkinsonism. However, despite dozens of studies indicating an ambiguous relationship between changes in striatal DA signaling and motor phenotype, this perseverating focus on striatum continues. Although DA release in substantia nigra (SN) was first reported almost 50 years ago, assessment of nigral DA signaling changes in relation to motor function is rarely considered. Whereas DA signaling has been well-characterized in striatum at all five steps of neurotransmission (biosynthesis and turnover, storage, release, reuptake, and post-synaptic binding) in the nigrostriatal pathway, the depth of such interrogations in the SN, outside of cell counts, is sparse. However, there is sufficient evidence that these steps in DA neurotransmission in the SN are operational and regulated autonomously from striatum and are present in human PD and aging and related animal models. To complete our understanding of how nigrostriatal DA signaling affects motor function, it is past time to include interrogation of nigral DA signaling. This brief review highlights evidence that changes in nigral DA signaling at each step in DA neurotransmission are autonomous from those in striatum and changes in the SN alone can influence locomotor function. Accordingly, for full characterization of how nigrostriatal DA signaling affects locomotor activity, interrogation of DA signaling in SN is essential.

## 1. Introduction

Ever since dopamine (DA) and norepinephrine (NE) neuronal pathways were identified and functionally characterized in vivo [1,2,3,4,5,6,7,8], the depth and breadth of studies of how these neurotransmitters affect both cognitive and motor behavior has been immense. The viability and function of the neuronal pathways that produce these neurotransmitters, nigrostriatal and ceruleo-cortical, respectively, are significantly decreased in Parkinson’s disease (PD). As such, the five components of neurotransmission (biosynthesis, storage, release, reuptake, and post-synaptic function) have been studied for respective contributions to deficits in DA or NE signaling in PD. The range of approaches used to interrogate these pathways include defining PD-related genes and physiological regulation of catecholamine genes [8,9,10,11,12], expression of catecholamine-regulating enzymes and transporters [13,14,15,16,17,18,19,20], post-translational modification of biosynthesis enzymes [21,22,23,24,25,26,27,28], neuron electrophysiological properties [29,30,31,32,33,34], release and uptake [35,36,37,38,39,40], pre- and post-synaptic receptor function [22,30,34,41,42,43,44,45], basal ganglia circuit function [46,47,48,49,50,51,52], and growth factor signaling [53,54,55,56,57,58,59,60,61,62,63,64]. Clearly the investment of resources in these multiple areas of research is for the ultimate goals of understanding PD etiology, the consequences of DA or NE loss that arise from PD on motor and cognitive skills, and to identify a sound mechanistic rationale for effective treatments to delay or arrest disease progression. Notably, the vast majority of studies that focus on the relationship between motor function and DA signaling have evaluated one or more of the five components of neurotransmission in the striatum, the terminal field region of the nigrostriatal pathway. It is important to keep in mind that at the time of PD diagnosis, the striatal regions already show ~70–80% loss of DA-regulating proteins or aspects of DA signaling (such as DA release). This non-linear relationship brings up two yet to be resolved questions: why is motor impairment not detected prior to 80% loss, and second, why does the severity of motor impairment continue to worsen when loss in striatum reaches near 100% 4–5 years after diagnosis [20].

## 2. Insights of How Striatal DA Signaling Affects Locomotor Function Have Reached a Plateau

In the context of PD, DA is, by far, the most studied of the catecholamines, with NE running a distant second. Since 1962, there have been ~29,000 publications associated with DA and PD vs. ~1700 associated with NE and PD. The evidence for deficient nigrostriatal DA signaling as the primary cause of motor symptoms of PD is strong. Yet there still remains a critical unresolved issue that hampers progress: a continuous perseverating focus to attribute deficient DA signaling in the striatum as the sole culprit for motor impairment. This focus is undoubtedly driven by the longstanding working model of basal ganglia circuit dysfunction that arises from the loss of striatal DA due to the progressive loss of nigrostriatal neurons. It is argued that this striato-centric focus has generated a plateau in our understanding of exactly how any of the five steps of neurotransmission with deficient DA-regulating function in striatum actually impair motor function. For definition purposes, the relation of nigrostriatal DA signaling to motor impairment will focus upon bradykinesia/hypokinesia, which is among four cardinal signs of PD which also include rigidity and postural instability and tremor at rest. Indeed, there are clinically based examples of where improvements in striatal DA signaling did not equate to alleviation of motor impairment in PD patients [60,64,65]. More evidence of this lack of alignment between striatal DA levels and severity of motor impairment is seen at the later stages of PD. Although the severity of motor impairment continues to worsen 4 to 5 years after PD diagnosis, loss of striatal DA-regulating proteins or signaling has already reached near 100% [20,66,67,68,69]. There is a comparable amount of evidence for this misalignment between striatal DA levels and motor function status in pre-clinical studies of rat PD models [22,57,58,59,70,71,72,73,74,75,76]. Motor impairment may also be present with far less than 80%, if any, striatal DA loss [54,65,70,72] or, conversely, motor impairment may not be present even though striatal DA loss meets or exceeds 80% [22,73,74]. Motor impairment can also be alleviated without any increase in or recovery of striatal DA or DA-regulating protein loss [54,57,59,73,74,75,76].

It is not the position of this review to assert that striatal DA signaling does not influence motor function. The weight of evidence that shows the influence of striatal DA signaling on basal ganglia circuits is too great to list here. However, the incongruities between the level of locomotor function and DA signaling in striatum can no longer be ignored if we are to solve which critical dopaminergic element(s) are to be targeted to maximize effective therapeutic strategies. This brief review will present evidence that challenges the central dogma that compromised DA signaling in striatum is the sole deficiency of DA that impairs locomotor function. The overwhelming evidence that nigrostriatal DA signaling does affect locomotor function has been obtained from our knowledge of PD and from studies that experimentally modulate components of DA neurotransmission. The key question is where in the nigrostriatal pathway does DA have the greatest influence on locomotor function; particularly regarding the mechanisms that drive the initiation of self-generated movement. Although the evidence that nigral DA signaling can influence motor function is sparse, it has nonetheless been in existence since the 1980s [77,78,79,80,81]. The paucity of studies evaluating the SN is likely due to a prevailing presumption that neurotransmitter functions at the axon terminal are the sole influence of behavioral outcomes. Thus, interrogation of nigral DA signaling has not been considered in experimental designs to define how components of nigrostriatal DA signaling affect locomotor activity. In this light, it is reasonable to presume that the numerous ambiguities between striatal DA regulation and motor function that have accumulated in the literature over the past several decades could have been resolved if assessment of nigral DA signaling was included in the study design.

## 3. Dissecting the Impact of the Five Components of DA Neurotransmission on Locomotor Function

As goes with the loss of nigrostriatal neurons in PD, the loss of DA-regulating proteins and processes involved in neurotransmission follows. Interference with the functions of any of these proteins or processes can also affect locomotor function in naïve (non-PD) animal models. Tyrosine hydroxylase (TH) is the rate-limiting step of DA biosynthesis, converting tyrosine to L-dihydroxyphenylalanine (L-DOPA). Inhibition of TH with alpha-methyl-p-tyrosine (AMPT) decreases DA tissue levels and inhibits locomotor activity [7,82,83,84,85,86]. In humans, inhibition of hyperkinetic movements, such as chorea, dystonia, or dyskinesia, can also be produced by AMPT [87,88]. The storage of DA and NE is controlled by vesicular monoamine transporter 2 (VMAT2), which imports monoamines like DA into synaptic vesicles using a proton gradient. This function is inhibited by reserpine, which also inhibits locomotor activity [89,90,91], as first identified by a parkinsonian symptom side effect produced in hypertension treatment [92]. VMAT2 is expressed in both striatum and SN [93,94], which confers the capacity for storing DA for eventual release in the entire nigrostriatal pathway.

Once DA is packaged in synaptic vesicles, it can be released by neuronal activity or by modulation of transporter function through stimulant action. At the extracellular level, DA release from the nigrostriatal pathway is the step that delivers tissue content, via vesicular delivery, to the synapse [95,96,97,98], wherein DA has four fates, binding to the pre- or post-synaptic DA receptors, reuptake into the neuron, or diffusion away from the release site [99]. Drugs that target DA receptors, the post-synaptic DA D_1_ receptor or pre- and post-synaptic DA D_2_ receptor, also influence locomotor activity and are targets for pharmacotherapy in PD treatment [100]. An acute regimen of antipsychotics such as haloperidol or either DA D_1_ or D_2_ receptor antagonists reduce locomotor activity [101,102,103,104,105]. Conversely, DA D_1_ or D_2_ agonists increase locomotor activity in rodents and primates [43,106,107] and improve motor functions in late-stage human PD [108,109,110]. The release of DA can also be modulated by DA D_2_ autoreceptor function [111] in both striatum and SN [31,112]. Finally, it should be mentioned that although the focus of this review on DA receptors is upon the D_1_ receptor, with brief overview of the D_2_ receptor, the three other DA receptors have been recently shown to play a role in locomotor impairments of PD, particularly the D_3_ and D_5_ receptors [113,114,115,116]. 

Functionally, the regulation of DA release by neuronal activity is critical for initiation of locomotor activity [117,118,119,120,121]. Deficits in DA release, such as occurs in aging or from over-expression of alpha-synuclein, are associated with decreased locomotor activity [122,123,124]. Conversely, under conditions that increase DA release, such as induced by amphetamine or methamphetamine [125,126,127], there is increased locomotor activity [128,129,130]. 

The termination of DA signaling occurs by reduction of extracellular DA levels in the synapse, largely, though not exclusively [99], through reuptake by the dopamine transporter (DAT) [131,132,133]; a process that occurs in SN as well as striatum [134,135,136]. DAT protein expression is considerably greater in the striatum [94], and, not withstanding possible influences of trafficking or contributions of other monoamine transporters, this difference may explain why DA release and uptake dynamics differ between these two regions [134,135,136]. Through constant trafficking between cytosol and plasma membrane, DAT function is dynamically regulated, including aging and in PD [137,138,139]. The DAT, like the DA D_2_ receptor, also has considerable interaction with other components of DA neurotransmission, including DA D_2_ receptors [34,112], and has considerable influence on maintaining DA tissue levels, TH expression, and phosphorylation selectively in the striatum, but not in SN [140,141]. There is also evidence of plasticity in DA uptake under conditions where DA and DAT levels are particularly low. In such cases, the NE transporter may also transport DA, with inherently low DA innervation or from severe loss of nigrostriatal neuron terminals [142,143]. 

Given the considerable influence of DAT on DA homeostasis, locomotor activity is strongly affected by DAT expression levels. DAT knockout mice or rats show a hyperkinetic phenotype [144,145,146]. This hyperkinetic phenotype is not likely explained by the low DA uptake capacity in the striatum due to DAT knockout, as DA tissue content levels are severely reduced to a level that is comparable to nigrostriatal lesion (>90% loss) [140,141]. Systemic delivery of nomifensine, a DAT inhibitor, increases locomotor activity [147], consistent with the hyperkinetic phenotype of the knockout [144,145,146]. While presumably this effect would be considered to be due to elevated extracellular DA levels in striatum from interference with DA uptake, we recently reported that infusion of nomifensine in striatum did not increase locomotor activity in aged rats, despite a striatum-specific increase in extracellular DA levels produced by nomifensine infusion therein [148]. 

## 4. Similarities and Differences in DA Signaling between Striatum and SN in Basal Conditions

Functional readouts of each of the five components of DA signaling have been established in the striatum and SN. These include expression levels of the regulating proteins (TH, DAT, VMAT2, and DA receptors), tissue and extracellular DA levels, DA release, DA uptake, and post-translational modifications of regulating proteins, particularly site-specific TH phosphorylation (Table 1). The differences in expression levels and function between striatum and SN under basal conditions provide the necessary basis upon which to evaluate the impact of perturbations on the nigrostriatal pathway that arise from aging and PD. At the biosynthesis level, several stark differences between striatum and SN are apparent. Tissue levels of DA are 20–30 times greater in the striatum than in the SN [22,54,84,94,148,149,150,151]. There are three factors that appear to drive this disparity in DA tissue levels: DA turnover, TH phosphorylation at ser31, and DAT expression. The SN has a 2-fold greater rate of DA turnover [22,54,84,94,148,149], which presumably means less available DA for release therein compared with striatum. The phosphorylation of TH at ser31, as opposed to ser40, matches the differences in DA tissue content across four DA regions in vivo [84,151]. TH phosphorylation stoichiometry at ser31 averages at least 3-fold higher in the striatum [18,22,54,84,137,141,148,149,150,151], which suggests a lesser capacity for DA biosynthesis in the SN. Finally, the expression of DAT and DA uptake capacity is much greater in the striatum than in SN [94,112,141,143]. In DAT knockout mice, there is severe loss of DA tissue levels in striatum, whereas there is no loss seen in the SN [141]. This disparity strongly suggests that striatal DA content is heavily influenced by DAT expression and function. Thus, it is clear that the disparities in DA biosynthesis (and catabolism), ser31 TH phosphorylation, DAT expression, and DA uptake between striatum and SN contribute to less tissue DA in the SN (Table 1). 

Additional observations of the other three components of DA neurotransmission indicate that storage capacity may be much greater in the SN, as VMAT2 expression with respect to inherent TH protein levels is much higher (Table 1) [94]. This differential storage capacity may counteract the great disparity in DA tissue levels between these two regions, as DA release capacity differences are not as great, with ~5-fold less DA release or extracellular levels in SN vs. striatum [112,134,136,152]. Finally, at the post-synaptic DA receptor level of DA neurotransmission, the few observations comparing striatum and SN within the same studies suggest a 30% greater expression level of the D_1_ receptor in the SN [148,149]. This differential may optimize DA signaling in the SN, particularly during loss of DA as would occur in PD progression. In summary, the current battery of results shows that differences in DA signaling between striatum and SN can be attributed to inherent differences in each of the five components of neurotransmission.

## 5. Approaches and Outcomes Needed to Discern Role of Striatal and Nigral DA Signaling

There is considerable evidence that the proteins and processes associated with the five steps of DA neurotransmission in the nigrostriatal pathway are operational in both striatum and SN. Modifications at these steps can alter DA signaling dynamics in either region, although there are notable differences in the functional dynamics between these regions at some of these steps, such as DAT expression and reuptake capacity [94,134,143]. The release of DA occurs in both striatum and SN with activation of nigrostriatal neurons [95,96,97,98,117,121,153] and is associated with self-directed movement [117,118,119,120,121,154]. Thus, with DA release contemporaneously occurring in striatum and SN, it would seem to be experimentally challenging to decipher the role of DA signaling in either region in locomotor function. However, with localized delivery of DA-modulating compounds into striatum or SN, it is plausible to target one or more of these steps in one region to modify and isolate DA signaling dynamics. Thus, interference at a step in DA neurotransmission in one region would be expected to influence extracellular DA levels only in the targeted region. Accordingly, this approach would at least partially address the reality of contemporaneous DA release that occurs in striatum and SN from neuronal activity. The critical outcomes needed from this approach are 2-fold: (1) modulate DA signaling in the targeted region, and (2) the modulation in the targeted area does not affect DA signaling in the non-targeted region. For example, to identify a role for nigral DA signaling in motor function, targeting a component of DA signaling in SN would be expected to not influence DA signaling in striatum. Such an approach is feasible, and therefore it is possible to parse out the relative contributions of DA signaling in striatum or SN and respective impact on locomotor function [44,54,84,121,148,149,155,156]. Most importantly, as the functional status of each step in DA neurotransmission is established in normal and disease states in either striatum or SN, region-specific modulation of DA signaling makes it possible to infer what impact the loss of such functions in disease states in these regions has on locomotor function.

## 6. Autonomy of DA Biosynthesis in SN and Impact on Motor Function in Aging and PD

Targeting one of the five steps of neurotransmission in a specific region of the nigrostriatal pathway represents an experimental approach to emulate specific mechanisms of DA signaling that exist in vivo in normal or disease states. For example, if TH levels are reduced selectively in the SN in a disease or aging model, then targeting TH activity in that region in an appropriate control animal can be useful to determine if the loss of TH or its function contributes to deficient DA signaling and locomotor function [149,155]. The specific targeting of SN or striatum to modulate DA signaling by targeting one of the five steps of neurotransmission is a critical experimental approach, because differences in DA regulation exist at multiple steps in normal (or naïve) rodents (Table 1), PD models, and in models of aging-related parkinsonism. Moreover, because such differences in multiple DA signaling steps between striatum and SN have also been identified in human PD and aging, it is feasible to determine, by experimental modulation within these regions, if any specific change in DA signaling is driving locomotor impairment.

Most studies that have evaluated how components of DA signaling from SN or striatum affect locomotor function have focused upon the DA biosynthesis step. This is likely due to the fact that there is severe TH loss in PD. Differences in TH expression, TH phosphorylation, and DA tissue content exist between the SN and striatum under normal [84,141,148,149,150,151,157,158], PD- [18,20,22,57,58,66,67,68,69,72,159], or aging-related conditions [54,61,84,148,149,151,159,160,161], both in animal models and in human PD [20,66,67,68,69,159] and aging [159,161,162,163]. As previously discussed and shown in Table 1, naïve (young age or without nigrostriatal lesion) rodents have 3- to 4-fold greater TH expression in the striatum. This difference in TH expression between striatum and SN is magnified by ~15- to 25-fold greater DA tissue levels in striatum; a disparity likely due to 3- to 10-fold greater ser31 TH phosphorylation in the striatum. Therefore, with the clear autonomy of TH regulation between striatum and SN, differences in TH expression or ser31 TH phosphorylation arising from nigrostriatal neuron loss or aging must be taken in the context of changes that are specific to each region. As such, determining the effect of nigrostriatal neuron loss or aging on these components of DA biosynthesis against changes in locomotor function must be evaluated with the understanding that baseline levels differ greatly between striatum and SN. These baseline levels contribute individually to the locomotor profile. Thus, a relatively smaller change in these components in one region, such as SN, may actually have a much more significant impact on locomotor function, despite a larger change observed in the other region, in the context of responses to perturbations in the nigrostriatal pathway from aging or PD.

## 7. Nigrostriatal DA Signaling and Aging-Related Parkinsonism: Relevance to PD

Bradykinesia (or hypokinesia) is the most prevalent motor symptom of aging-related parkinsonism. As shown in rat models of aging-related parkinsonism and PD, three indices of DA biosynthesis (DA tissue content, TH protein, and TH phosphorylation) in the SN, but not striatum, are associated with changes in locomotor function. From the standpoint of aging, studies on rodents [54,84,122,123,148,149,150,151], primates [164,165,166], and humans [167,168,169] all indicate that loss of DA or TH in striatum varies considerably, from virtually no loss to 50% compared to young cohorts. Notably, no aging study has reported that striatal DA or TH loss reaches the accepted 80% loss threshold associated with PD motor symptom onset [19,20,73,170,171,172]. However, in primates, the severity of bradykinesia covaries with TH or DAT loss in the SN [160]. Moreover, in an established rat model of aging, nigra-specific loss of TH protein and decreased ser31 TH phosphorylation were also associated with a 40% decrease in DA tissue levels, with no loss of DA, TH protein, or decreased ser31 phosphorylation in striatum [151]. To determine if this nigra-specific loss of DA was contributing to decreased locomotor activity (which would be bradykinesia/hypokinesia in humans), we infused the TH inhibitor AMPT into the SN of young rats to produce DA reduction comparable to that in aged rats. This delivery in the SN did not affect DA levels in striatum. Locomotor activity was decreased during the time established for DA reduction in the SN [155]. In another study, we targeted the striatum with AMPT to decrease DA by 30% (the maximum obtainable by this approach). Although DA reduction was specific for the striatum, there was no effect on locomotor activity [149].

Using the same approach in aging rats, we infused nomifensine into either region to determine if augmenting extracellular DA levels, by blocking DA reuptake, would increase locomotor activity [148]. Essentially, this approach was to counteract aging-related diminished DA release that was previously established to occur in either region [122,123,164]. Again, the infusion approach produced a region-specific increase in extracellular DA levels. We found that increasing DA by nomifensine infusion into the SN was associated with increased locomotor activity, whereas nomifensine infusion into the striatum by nomifensine had no effect on locomotor activity [148]. These results indicate that aging-related decreases in DA release in the nigrostriatal pathway associated with decreased locomotor activity are due to decreased release in the SN. Thus, by experimentally modulating DA locally in SN or striatum to mimic or counteract aging effects at the biosynthesis or (indirectly) release steps, the results point to deficient DA signaling in the SN as a contributing mechanism to reduced locomotor activity in aging rats. It would be logical therefore to presume that the inhibition of motor activity following systemic AMPT [7], or the enhancement of motor activity following systemic nomifensine [122,147] or elimination of reuptake in the DAT knockout [144,145,146], is being driven, at least in part, by modulation of nigral DA signaling.

**Figure 1:**
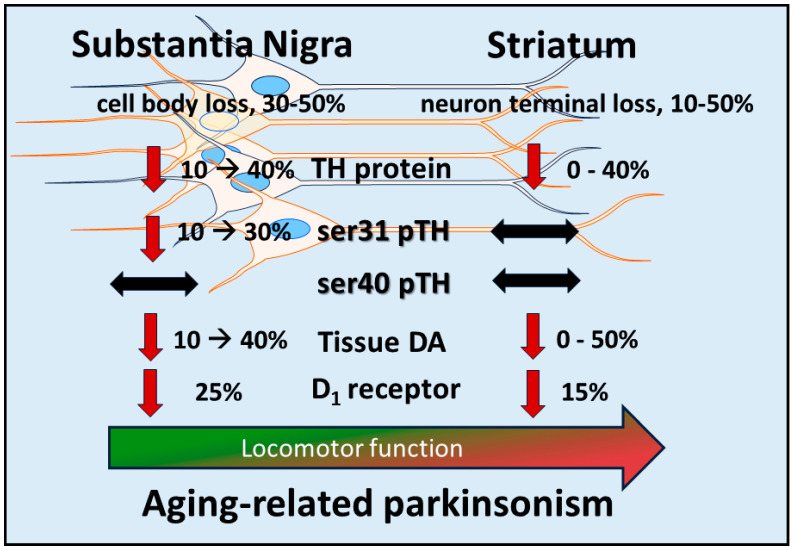
Molecular changes in components of dopamine (DA) signaling in substantia nigra (SN) are autonomous from those in striatum during aging and coincide with decreasing locomotor function. Unlike PD, loss of tyrosine hydroxylase (TH) protein and tissue DA in striatum is substantially less and more highly variable (-) in aging, with maximum loss of ~50% being the most ever reported [164,165,166,167,168,169]. Conversely, in the SN, there are several aging-related changes occurring at the biosynthesis and receptor levels. Loss of the DA D_1_ receptor (D_1_) occurs in the middle to late-middle stages of the lifespan and temporally coincides with the onset of locomotor decline [149]. Loss of TH protein progresses (→) in the SN toward the latter (aged) part of the lifespan, with steadily decreasing DA tissue content [84,148,151,159,160,161,162,163,164,165,166]. Notably, there is also a decrease in site-specific TH phosphorylation at ser31 (ser31 pTH), but not at ser40 (ser40 pTH), that occurs only in the SN [151]. Aging-related loss of nigral TH protein and tissue DA is comparable to loss at the onset of locomotor impairment in PD [19], suggesting that DA tissue losses from decreased ser31 TH phosphorylation and TH protein are mechanisms of hypokinesia seen in aging.

To summarize, aging-related parkinsonism cannot be explained by loss of TH protein or DA tissue levels in striatum, given the high variability of loss across studies and that TH or DA loss does not reach the accepted consensus of 80% loss associated with the onset of motor symptoms in PD [19]. Instead, the deficiencies in DA signaling of the nigrostriatal pathway to drive parkinsonism reach sufficient levels in the SN, but not in striatum (Figure 1). Thus, even though parkinsonian motor symptoms occur during aging, the considerable variability in DA or TH loss in the striatum makes it impossible to pin culpability on deficient striatal DA signaling as the single source of aging-related parkinsonian signs. Our work, along with others, makes the case that multiple steps of DA neurotransmission in the SN are affected in aging that coincide with the development of parkinsonian-like symptoms. The first likely event in the lifespan is an aging-related decrease in DA D_1_ receptors (to be discussed further below) followed by decreased expression of TH protein and a phosphorylation-site-specific decrease in ser31 (and not ser40). It is unknown if the decrease in TH protein is due only to neuronal loss that has been documented to also occur [162]. As a result of these decreases, DA tissue content is reduced, likely driving the decrease in DA release previously reported in the SN [164]. Importantly, the decreases in TH protein, neuron loss, and tissue DA in the SN in models of aging are comparable to those reported in the SN in human PD and PD models at the onset of bradykinesia [19,20,35,159,170,171]. This consistency with the changes in the SN that also occur in PD makes it further plausible that deficient DA signaling in the SN is responsible for decreased locomotor activity or parkinsonism in aging.

## 8. Nigrostriatal DA Signaling and PD-Related Motor Impairment

From the perspective of deficient DA signaling impact on motor impairment in PD, a long-standing unresolved issue is why motor impairment does not occur until there is 70–80% TH or DA loss in striatum. It was long thought that increased DA turnover reflected increased DA signaling during progressive loss of the nigrostriatal neuron terminals [19,73,172,173,174,175], thus compensating for TH protein loss to enable normal locomotor activity. L-DOPA, the product of TH, remains the gold standard for treating motor symptoms. Thus, it stands to reason that compensating for TH loss through engagement of innate compensatory mechanisms that increase DA levels would promote maintaining locomotor function until striatal TH loss was too severe.

Increased DA turnover was proposed to be an indicator of enhanced DA signaling to compensate for TH protein loss during nigrostriatal neuron loss [19,52,73,172,173,174,175]. However, Bezard and colleagues definitively showed in an elegant timeline study using MPTP-lesioned primates in which increased DA turnover occurred only after bradykinesia manifested; there was no evidence of increased DA turnover during the asymptomatic period [19]. Also, 80% TH and DA loss in striatum appeared to be necessary for the onset of bradykinesia; even 60% TH loss in striatum was observed during the asymptomatic period. Fortunately, this study also assessed TH loss in the SN and found that, at the onset of motor impairment, there was ~40% loss in the SN; far less than 80% loss seen at the axon terminals. This loss in the SN may be related to regionally selective loss of nigral neurons, as shown in human aging and PD [159,161]. Also, this disparity in TH loss between SN and striatum has strong translational relevance because this disparity consistently manifests in human PD [20,66,67,68]. Nonetheless, the lack of evidence to support a role for increased DA turnover in striatum to offset the onset of locomotor impairment gave rise to the consideration that non-DA related mechanisms to be responsible for delaying the onset of motor impairment [52].

**Figure 2:**
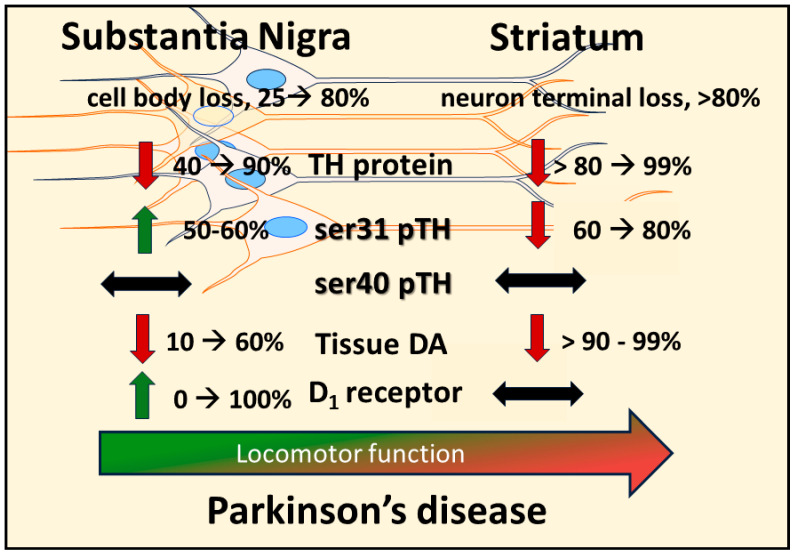
Disparate molecular changes in dopamine (DA) signaling components in substantia nigra (SN) and striatum in response to neuronal loss and relation to decreased locomotor function. Induction of nigrostriatal neuron loss by 6-OHDA produces a progressive loss of neurons over 4 weeks. Loss of tyrosine hydroxylase (TH) protein in SN is less than the magnitude of loss in the striatum at the earlier time points post-lesion, and tissue DA loss is substantially and consistently less in the SN than in striatum. In response to TH loss, there is a site-specific increase in TH phosphorylation at ser31 (ser31 pTH), not ser40 (ser40 pTH), restricted to the SN; whereas in striatum, there is a progressive decrease in ser31 pTH. This increase in ser31 pTH in the SN offsets the progressive loss of TH therein to keep DA loss at a lower level than TH. As DA tissue loss increases in the SN, the DA D_1_ receptor (D1) increases expression at the latter stages of neuron loss. The increases in both ser31 TH phosphorylation and D1 in the SN are compensatory mechanisms to delay the onset of locomotor impairment and alleviate its severity.

Recent work from our group indicates that the compensatory mechanism to mitigate the severity of hypokinesia and delay its onset against progressive nigrostriatal neuron loss is related to increased DA signaling in the SN, and not striatum [22]. This mechanism involves an increase in ser31 TH phosphorylation, specifically in the SN, that begins early after nigrostriatal loss induction by 6-hydroxydopamine (6-OHDA) and is maintained at least until neuronal loss reaches 80% in the SN. As a result of this increase in ser31 TH phosphorylation, there is less loss of DA as compared to TH throughout neuronal loss [22]. This differential in DA and TH loss also manifests in the SN, contralateral to the lesioned side, as TH loss begins there at a later time after lesion induction. When correlating the loss of DA in SN and striatum against the severity of motor decline in the open field, only DA loss in the SN has significant correlation [22]. In striatum, we found no difference in TH and DA loss, as both exceeded 90% early after lesion induction, commensurate with decreased ser31 TH phosphorylation and increased DA turnover. In contrast, DA turnover decreased in the SN as neuron loss progressed. Our findings of diminished lesion impact on DA tissue content in the SN are also reflected in the extracellular realm, wherein baseline DA levels are unaffected by 6-OHDA lesions despite severe neuronal loss [176]. Together, these results frame a new perspective on the mechanism by which motor impairment is delayed by increased DA biosynthesis in the SN, despite progressive nigrostriatal neuron loss that occurs in PD (Figure 2). Moreover, these results are disease-relevant and further support a role for nigral DA signaling in locomotor function.

## 9. Autonomy of Post-Synaptic DA Signaling in SN and Impact on Motor Function

**Figure 3:**
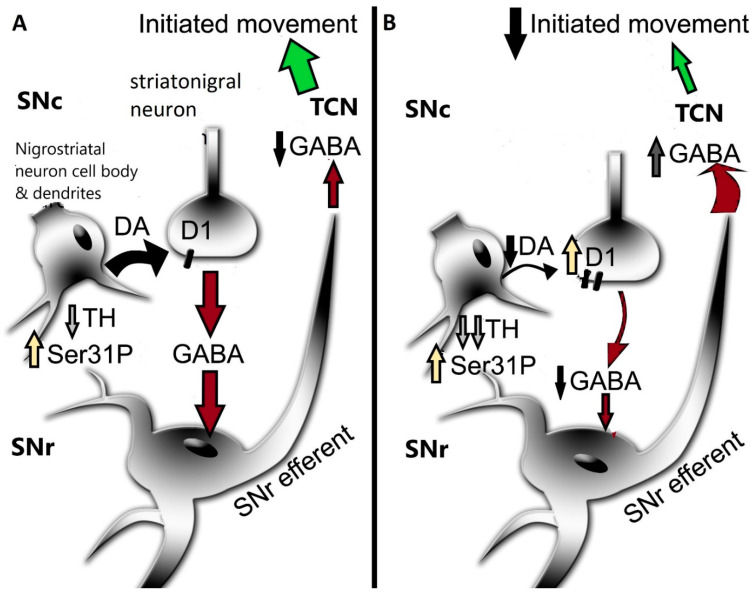
Compensatory response in substantia nigra (SN) to maintain dopamine (DA) signaling. (**A**) Early stage of nigrostriatal neuron loss. During nigrostriatal neuron loss, tyrosine hydroxylase (TH) protein loss (downward arrow) in SN precedes neuron loss. To maintain DA tissue levels, TH phosphorylation at ser31 (Ser31P) increases (beige upward arrow), offsetting DA loss that would otherwise occur (as seen in striatum) [22]. Adequate tissue DA levels maintain sufficient DA release in SN pars compacta (SNc) to activate post-synaptic DA D_1_ receptors (D_1_), thus enabling GABA release (red arrows) from striatonigral terminals. This release from striatonigral neurons in SN pars reticulata (SNr) mitigates tonic GABA release from the SNr efferent on the thalamocortical neurons (TCN) to promote locomotor activity via glutamate release (green arrows). (**B**) Late stage of nigrostriatal neuron loss. Although ser31P is still increased, TH protein progresses further and is sufficient to diminish DA tissue levels, although DA loss is still less than TH protein loss [22]. The decrease in DA tissue content diminishes release capacity. In response, the D1 is upregulated (beige upward arrow) on post-synaptic striatonigral terminals to compensate for decreased synaptic DA levels. The overall plasticity of increased DA biosynthesis and D_1_ expression in the SN is hypothesized to mitigate the severity of bradykinesia/hypokinesia that would be expected from severe TH protein and nigrostriatal neuron loss.

The activation of the DA D_1_ receptor, expressed on striatonigral neurons, in the SN mediates GABA release [30,42,47]. This release of GABA decreases the normally inhibitory output of the basal ganglia, thus reducing the inhibitory output that facilitates the generation of movement. Both aging and PD can affect D_1_ receptor expression, which posits the function and expression of this receptor as contributing mechanisms in motor impairment. In the middle to late-middle stage of the lifespan, there is a 30% decrease in expression of this receptor in the rat SN and a smaller decrease in striatum [149] (Figure 1). This decrease is associated with an aging-related decrease in locomotor activity. In human aging, DA D_1_ receptor expression also decreases proportionally with age [177], which may contribute to the onset of mild bradykinesia beginning in late middle age in humans. Previous work by Trevitt and colleagues modulated D_1_ receptor function in SN and striatum to evaluate its relative impact on locomotor function in rats. They showed nigral infusion of a DA D_1_ receptor antagonist was highly potent in reducing operant behavior and open-field activity [44]. Decreased locomotor activity is also produced by DA D_1_ receptor antagonists following systemic delivery [105]. Thus, it is plausible that the locomotor-modulating action of DA D_1_ receptor drugs, in animal models and humans alike, is driven by modulation of its post-synaptic functions in the SN [43,103,105,106,107,108,109]. Thus, it is plausible that local release of GABA in the SN depends largely upon activation of DA D_1_ receptors following local DA release in the SN [30,42,47]. With the understanding that GABAergic input to the midbrain arises from nuclei, in addition to striatum, that influence locomotor activity [178], GABA release from striatonigral terminals enables the disinhibition of basal ganglia output from the SN *pars reticulata* neurons. It is feasible that this sequence of events, that originates from DA release in the SN, provides the signal to increase locomotor activity (Figure 3). This work also suggests that the first onset of aging-related decreases in locomotor activity in the lifespan may be driven by decreased DA D_1_ receptor expression in the SN [149]. Consistent with this relationship, aging-related deficits in motor function may be alleviated by increased DA D_1_ receptor expression, exclusively in the SN [148].

In PD, the DA D_1_ receptor has recently been identified as a novel target to treat motor impairment in the later stages of the disease [108,109,110]. The status of DA D_1_ receptor expression or function is far less known than the DA D_2_ receptor [100,179]. Our work in the 6-OHDA model indicates that the DA D_1_ receptor is upregulated, specifically in the SN, as nigrostriatal neuron and DA loss increase therein [22]. In contrast to the changes in SN, D_1_ receptor expression is unchanged in striatum, despite the severe loss of DA therein beginning early after nigrostriatal neuron lesions. We speculate this increase in the SN is a response by the striatonigral neurons to maintain DA signaling in the SN. Notably, D_1_ receptor expression does not change in SN in the early stages of neuronal loss, when DA tissue levels are unaffected. Thus, with D_1_ receptor upregulation in the latter stages of PD, it stands to reason that a D_1_ receptor agonist could substitute for DA, given the reduction in DA levels at the latter stage of neuron loss.

In summary, multiple lines of evidence from human PD and aging and related animal models indicate that DA signaling in the SN plays a significant role in locomotor activity levels. Changes at the biosynthesis, release, reuptake, and post-synaptic signaling steps in the SN occur autonomously from changes (if any) in the striatum, making a clear case that augmenting DA signaling in the SN alone could be achieved by several possible strategies to alleviate locomotor impairment. Moreover, targeting specific steps of DA neurotransmission that are affected in aging and PD can reveal which deficit (and where in the nigrostriatal pathway) is responsible for decreasing DA signaling to impair locomotor activity. As long as there is a means to locally modulate one or more of the components of DA neurotransmission, such as inhibition of DA biosynthesis in striatum or SN [84,148,149,155] or augmenting it by infusion of L-DOPA [39,180,181,182], it is possible to pinpoint the most critical losses responsible for locomotor impairment.

## 10. Upstream Regulators of DA Signaling: The Role of GDNF Signaling in SN

There has been a great need to find treatment for PD that is disease modifying, in addition to a therapeutic approach that can reduce the amount of L-DOPA needed to maintain mobility without debilitating side effects such as L-DOPA-induced dyskinesia. In the 1990s, glial-cell-line-derived neurotrophic factor (GDNF) emerged as a top candidate for treatment of motor impairment in PD based upon encouraging pre-clinical studies in rodent and non-human primates [56,57,58,59]. Notably, GDNF had the rather remarkable attribute of long-term impact on constituents of DA signaling (such as increased DA tissue content and ser31 TH phosphorylation), particularly in the SN, after a single delivery [56,57,58,61,62,183]. These long-term effects of GDNF were eventually revealed in clinical trials, wherein motor benefits to patients endured for up to a year following discontinuation [184,185] and motor benefits were realized while receiving GDNF [55,186]. In pre-clinical rat PD models, this long-term effect of GDNF may be driven by increased expression of its receptor, GFR-α1, specifically in the SN [53,54,187]. Notably, GFR-α1 itself alleviates TH and DA loss after 6-OHDA lesions in the SN, but not striatum [187], and can increase TH and DA levels, selectively in the SN, with increased locomotor activity, in aged rats [54].

More recent clinical trials with GDNF reported failure to reach the primary end point of improvement in motor scores in GDNF recipients relative to placebo control groups [60,64], leading the field to reconsider its therapeutic potential for treating the motor impairments of PD [188]. It should be briefly noted that in the failed trials there was evidence of increased DA signaling in the putamen [60,64]; an outcome representing more evidence of the ambiguity between striatal DA signaling and locomotor function. Retrograde transport of GDNF from striatum to the SN has been a well-documented physiological event [62,189,190,191]. Given the impact of GDNF or GFR-α1 in the SN on DA signaling and strong association with improved locomotor activity, it is likely that the trophic action of GDNF depends upon there being sufficient GFR-α1 levels in both striatum (for retrograde transport) and in the SN wherein the stimulating effects on DA signaling can occur [53,54,58,61,192]. The lack of GFR-α1 has been recently identified as a potential major challenge, as GFR-α1 expression progressively decreases in DA neurons as neuronal loss proceeds [63].

## 11. Conclusions

We have known for nearly 50 years that DA is released from the somatodendritic region of nigrostriatal neurons in the SN [193,194] and that the five steps of DA neurotransmission that comprise DA signaling (biosynthesis, storage, release, uptake, and post-synaptic receptor activation) in striatum are also present, functional, and targetable in the SN. Moreover, substantial evidence shows that DA signaling is autonomously regulated in SN from striatum. Thus, it cannot be assumed that changes in DA signaling in one compartment are also occurring in the other compartment. Therefore, under physiological conditions, despite that DA release occurs in both striatum and SN during neuron activation, modulation at specific steps of DA neurotransmission in one of these two regions can alter the magnitude of DA release capacity or post-synaptic function in only one region. Given the multiple examples of studies that have shown incongruity between components of striatal DA signaling and locomotor function, it stands to reason that changes in DA signaling in the SN in these studies could have been the culpable mechanism.

As a final point of consideration, one aspect of this review that bears mentioning is that the focus has been on protein expression and the respective functions at the five steps of neurotransmission. Governing the expression and function of proteins at these steps is nigrostriatal neuronal integrity. The numbers of DA terminals and somatodendritic components undoubtedly have significant, and ultimate, influence on synaptic function and, indeed, motor function [35,66,69,93,135,160,162]. Neuron viability is moderately affected in aging [160,161,162,163] with markers of DA function largely intact [164,165,166]. However, this stands in contrast to PD, wherein terminal functions are drastically reduced and eventually eliminated by 5 years after diagnosis [20]. From the perspective of whether DA terminals or evidence of DA function in striatum are required for adequate, or at least improved, motor function, the answer is, perhaps, no [22,55,57,58,60,64,65]. Noting the remarkable plasticity of DA function during nigrostriatal neuron loss [18,22,24,26,39,85,143,174,175,176], it should be clear by now that investigations of the source of motor impairment (or improvements) should extend well beyond the status of terminal field status and neuronal viability.

Given the autonomy of DA regulation between striatum and SN, changes in nigral DA signaling alone theoretically could influence locomotor function, and the evidence for this continues to increase. Indeed, although there is a substantially smaller number of studies of interrogating nigral DA signaling, and an even smaller number of studies that also measure locomotor activity against it, there is congruity with the direction of change in nigral DA modulation and locomotor activity in a number of studies [44,54,71,78,79,80,148,149,151,155,156]. These results are also consistent with studies reporting changes in basal ganglia output from the SN as a result of modulating DA signaling specifically in the SN [30,47,195,196,197,198]. These results are applicable in PD and aging, as the autonomy of DA signaling and components of DA neurotransmission exist at multiple levels [18,19,20,22,35,36,66,67,68,69,84,149,150,151,155,156,157,158,167,168]. This has direct implications when identifying whether the striatum or SN is the source of DA signaling deficits that drive locomotor impairment and its severity in both aging and PD [199].

## 12. Future Directions

The loss of nigrostriatal neurons in PD has paved the way in our understanding how DA loss affects motor function and, in general, how changes in DA signaling components affect locomotor function. However, it is past time to consider that the continuing loss of DA signaling components remaining in the SN only a few years after diagnosis may well be driving the worsening locomotor impairment in PD patients. Moreover, molecular changes in DA signaling in SN may be particularly viable therapeutic targets to delay motor impairment in the prodromal phase of PD [22,52,200,201,202]. Therefore, a collective consensus in recognizing the role of nigral DA signaling in locomotor function will expand our understanding of the mechanisms, including those upstream of DA (such as GDNF signaling), that contribute to locomotor impairment and its restoration. For example, with evidence for DA compensation occurring in the SN to mitigate the severity of locomotor decline, the inherent mechanisms driving it may represent targets to maintain locomotor function when TH protein loss is too great. It will also be important to delve further into understanding what striatal DA signaling is doing for maintaining locomotor function. For example, tremor at rest is a cardinal sign in PD. However, in aging-related parkinsonism, the evidence for its presence is scarce; notably, TH and DA loss are nowhere near the severity that occurs in PD. Therefore, DA deficits in striatum may reach a level of severity that promotes this involuntary movement only in PD. Finally, it should be a priority to determine what compartment of the nigrostriatal pathway should be targeted to maximize the efficacy of potential treatments, such as GDNF, on locomotor recovery. The potential for increased nigral DA signaling as a mechanism for locomotor recovery should stand as a priority comparable to the attention that the striatum has garnered.

## Figures and Tables

**Table 1:** Comparison of dopamine (DA) regulation between striatum and substantia nigra (SN) at each of the 5 steps in neurotransmission. **(1) DA biosynthesis**, 6 different measures are presented: tissue DA (as ng DA/mg tissue protein), DA turnover (ng dihydroxyphenylacetic acid per ng dopamine), tyrosine hydroxylase (TH) (as ng TH protein/µg tissue protein), DA normalized against total TH protein (as ng DA/ng TH, an estimate of DA biosynthesis), ser31 and ser40 TH phosphorylation stoichiometry (ser31, ser40) (determined as ng phosphorylation quantity per ng total TH protein); **(2) DA uptake**, 3 different measures are presented: DAT per TH (total DAT immunoreactivity per total TH protein quantified), DAT per protein (total DAT immunoreactivity per total protein), and DA uptake (as pmole DA per second or per total protein per minute); **(3) DA storage**, 2 different measures are presented: VMAT per TH (vesicular monoamine transporter 2 (VMAT) per total TH protein) and VMAT per protein (VMAT per total protein); **(4) DA release or baseline levels**, results presented as µM concentration during release or fmole quantity at baseline *; **(5) post-synaptic DA receptor D1R**, results presented as immunoreactivity of D1R quantified per total protein.

DA Biosynthesis								DA Uptake		DA Storage		DA	Post-Synaptic	
Region	ng/mg	DA	ng/µg				DAT	DAT	DA Uptake	VMAT	VMAT	Release	DA Receptor	
	DA	Turnover	TH	ng DA/ng TH	ser31	ser40	per TH	Per Protein	(pmole/unit time)	per TH	Per Protein	or Baseline *	D1R	Study
**Striatum**	214		0.391	0.547	0.33	0.022								Salvatore et al. [151]
	225	0.068	1.06	0.212	0.322	0.026	26	1637		17	6.1			Keller et al. [94]
	128	0.016	0.6	0.213	0.29	0.025								Salvatore and Pruett [84]
									7.1 pmole/mg			1.14 µM [134]		Chotibut et al., 2012 [143]
									protein/min [139]			1.0 µM [112]		Hoffman et al., 1998 [134]
									5.0 pmole/s [130]			5.1 fmol [152] *		Cragg and Greenfield, 1997 [112]; Robertson et al., 1991 [152]
	318	0.08	0.53	0.6	0.37									Pruett and Salvatore, 2013 [54]
	225		0.52	0.433	0.635 *	0.064 *								Salvatore et al., 2016 [150]
	140	0.19	0.45	0.311	0.31								55	Salvatore et al., 2017 [149]
	225	0.11	0.48	0.469	0.26	0.026		3772					70	Salvatore et al., 2023 [148]
	150	0.11	0.4	0.375	0.38	0.015								Kasanga et al., 2023 [22]
MEAN	**203.1**	0.096	**0.554**	**0.395**	**0.323**	0.023	**26**	**2704.5**	**6**	17	**6.1**	**1.1**	62.5	
SEM	58.3	0.053	0.202	0.135	0.039	0.004	0	1067.5		0	0			
**SN**	7.6		0.053	0.143	0.104	0.046								Salvatore et al. [151]
	9.5	0.15	0.15	0.063	0.108	0.053	2.6	197	110		5			Keller et al. [94]
	5.9	0.2	0.09	0.066	0.069	0.016								Salvatore and Pruett [84]
									3.3 pmole/mg			0.18 µM [134]		Chotibut et al., 2012 [143] Hoffman et al., 1998 [134]
									protein/min [139]			0.25 µM [112]		Cragg and Greenfield, 1997 [112]
									1.0 pmole/s [130]			0.61 fmol [152] *		Robertson et al., 1991 [152]
	9.5	0.15	0.051	0.186	0.09									Pruett and Salvatore, 2013 [54]
	6.5		0.023	0.283	0.144 *									Salvatore et al., 2016 [150]
	6	0.22	0.06	0.1	0.134	0.049		82						Salvatore et al., 2017 [149]
	8.5	0.29	0.049	0.173	0.14	0.022	828	100						Salvatore et al., 2023 [148]
	10	0.2	0.12	0.083	0.09	0.018								Kasanga et al., 2023 [22]
MEAN	7.9	**0.202**	0.075	0.137	0.105	0.034	2.6	512.5	2.1	**110**	**5**	0.22	**91**	
SEM	1.6	0.047	0.04	0.071	0.023	0.016	0	315.5		0	0			

## Data Availability

Research results are available upon reasonable written request.

## References

[1] Glowinski J., Axelrod J., Iversen L.L. (1966). Regional studies of catecholamines in the rat brain. IV. Effects of drugs on the disposition and metabolism of H3-norepinephrine and H3-dopamine. J. Pharmacol. Exp. Ther..

[2] Glowinski J., Iversen L.L. (1966). Regional studies of catecholamines in the rat brain. I. The disposition of [3H]norepinephrine, [3H]dopamine and [3H]dopa in various regions of the brain. J. Neurochem..

[3] Axelrod J. (1971). Noradrenaline: Fate and Control of its biosynthesis. Science.

[4] Thierry A.M., Blanc G., Sobel A., Stinus L., Glowinski J. (1973). Dopaminergic terminals in the rat cortex. Science.

[5] Coyle J.T., Axelrod J. (1971). Development of the uptake and storage of L-[3H] norepinephrine in the rat brain. J. Neurochem..

[6] Carlsson A., Dahlstroem A., Fuxe K., Lindqvist M. (1965). Histochemical and biochemical detection of monoamine release from brain neurons. Life Sci..

[7] Anden N.E., Carlsson A., Dahlstroem A., Fuxe K., Hillarp N.A., Larsson K. (1964). Demonstration and mapping out of nigro-neostriatal dopamine neurons. Life Sci..

[8] Rech R.H., Borys H.K., Moore K.E. (1966). Alterations in behavior and brain catecholamine levels in rats treated with alpha-methyltyrosine. J. Pharmacol. Exp. Ther..

[9] Nagatsu T., Nakashima A., Ichinose H., Kobayashi K. (2019). Human tyrosine hydroxylase in Parkinson’s disease and in related disorders. J. Neural Trans..

[10] Kumer S.C., Vrana K.E. (1996). Intricate regulation of tyrosine hydroxylase activity and gene expression. J. Neurochem..

[11] Reed X., Bandrés-Ciga S., Blauwendraat C., Cookson M.R. (2019). The role of monogenic genes in idiopathic Parkinson’s disease. Neurobiol. Dis..

[12] Nishioka K., Imai Y., Yoshino H., Li Y., Funayama M., Hattori N. (2022). Clinical Manifestations and Molecular Backgrounds of Parkinson’s Disease Regarding Genes Identified from Familial and Population Studies. Front. Neurol..

[13] Chotibut T., Davis R.W., Arnold J.C., Frenchek Z., Gurwara S., Bondada V., Geddes J.W., Salvatore M.F. (2014). Ceftriaxone increases glutamate uptake and reduces striatal tyrosine hydroxylase loss in 6-OHDA Parkinson’s model. Mol. Neurobiol..

[14] Pickel V.M., Beckley S.C., Joh T.H., Reis D.J. (1981). Ultrastructural immunocytochemical localization of tyrosine hydroxylase in the neostriatum. Brain Res..

[15] Moore C., Xu M., Bohlen J.K., Meshul C.K. (2020). Differential ultrastructural alterations in the Vglut2 glutamatergic input to the substantia nigra pars compacta/pars reticulata following nigrostriatal dopamine loss in a progressive mouse model of Parkinson’s disease. Eur. J. Neurosci..

[16] Fiorenzato E., Antonini A., Bisiachhi P., Weis L., Biundo R. (2021). Asymmetric Dopamine Transporter Loss Affects Cognitive and Motor Progression in Parkinson’s Disease. Mov. Disord..

[17] Beauchamp L.C., Dore V., Villemagne V.L., Xu S., Finkelstein D., Barnham K.J., Rowe C. (2023). Utilizing 18F-AV-133 VMAT2 PET Imaging to Monitor Progressive Nigrostriatal Degeneration in Parkinson Disease. Neurology.

[18] Salvatore M.F. (2014). ser31 tyrosine hydroxylase phosphorylation parallels differences in dopamine recovery in nigrostriatal pathway following 6-OHDA lesion. J. Neurochem..

[19] Bezard E., Dovero S., Prunier C., Ravenscroft P., Chalon S., Guilloteau D., Crossman A.R., Bioulac B., Brotchie J.M., Gross C.E. (2001). Relationship between the appearance of symptoms and the level of nigrostriatal degeneration in a progressive 1-methyl-4-phenyl-1,2,3,6-tetrahydropyridine-lesioned Macaque model of Parkinson’s disease. J. Neurosci..

[20] Kordower J.H., Olanow C.W., Dodiya H.B., Chu Y., Beach T.G., Adler C.H., Halliday G.M., Bartus R.T. (2013). Disease duration and the integrity of the nigrostriatal system in Parkinson’s disease. Brain.

[21] Perez R.G., Waymire J.C., Lin E., Liu J.J., Guo F., Zigmond M.J. (2002). A Role for alpha -Synuclein in the Regulation of Dopamine Biosynthesis. J. Neurosci..

[22] Kasanga E.A., Han Y., Shifflet M.K., Navarrete W., McManus R., Parry C., Barahona A., Nejtek V.A., Manfredsson F.P., Kordower J.H. (2023). Nigral-specific increase in ser31 phosphorylation compensates for tyrosine hydroxylase protein and nigrostriatal neuron loss: Implications for delaying parkinsonian signs. Exp. Neurol..

[23] Johnson M.E., Salvatore M.F., Maiolo S.A., Bobrovskaya L. (2018). Tyrosine hydroxylase as a sentinel for central and peripheral tissue responses in Parkinson’s progression: Evidence from clinical studies and neurotoxin models. Prog. Neurobiol..

[24] Shehadeh J., Double K.I., Murphy K.E., Bobrovskaya L., Reyes L., Dunkely P.R., Halliday G.M., Dickson P.W. (2019). Expression of tyrosine hydroxylase isoforms and phosphorylation at serine 40 in the human nigrostriatal system in Parkinson’s disease. Neurobiol. Dis..

[25] Nakashima A., Mori K., Kaneko Y.S., Hayashi N., Nagatsu T., Ota A. (2011). Phosphorylation of the N-terminal portion of tyrosine hydroxylase triggers proteasomal digestion of the enzyme. Biochem. Biophys. Res. Commun..

[26] Kolacheva A., Alekperova L., Pavlova E., Bannikova A., Ugrumov M.V. (2022). Changes in tyrosine hydroxylase activity and dopamine synthesis in the nigrostriatal system of mice in an acute model of Parkinson’s disease as a manifestation of neurodegeneration and neuroplasticity. Brain Sci..

[27] Haycock J.W., Haycock D.A. (1991). Tyrosine hydroxylase in rat brain dopaminergic nerve terminals: Multiple-site phosphorylation in vivo and in synaptosomes. J. Biol. Chem..

[28] Morgenroth V.H., Hegstrand L.R., Roth R.H., Greengard P. (1975). Evidence for involvement of protein kinase in the activation by adenosine 3′:5′-monophosphate of brain tyrosine 3-monooxygenase. J. Biol. Chem..

[29] Willard A.M., Islett B.R., Whalen T.C., Mastro K.J., Ki C.S., Mao X., Gittis A.H. (2019). State transitions in the substantia nigra reticulata predict the onset of motor deficits in models of progressive depletion in mice. eLife.

[30] Kliem M.A., Maidment N.T., Axkerson L.C., Chen S., Smith Y., Wichmann T. (2007). Activation of nigral and pallidal dopamine D1-like receptors modulates basal ganglia outflow in monkeys. J. Neurophysiol..

[31] Dagra A., Miller D.R., Lin M., Gopinath A., Shaerzadeh F., Harris S., Sorrentino Z.A., Stoier J.F., Velasco S., Azar J. (2021). α-Synuclein-induced dysregulation of neuronal activity contributes to murine dopamine neuron vulnerability. NPJ Park. Dis..

[32] Matschke L.A., Komadowski M.A., Stohr A., Lee B., Henrick M.T., Griesbach M., Rinne S., Geibl F.F., Chiu W.H., Koprich J.B. (2022). Enhanced firing of locus coeruleus neurons and SK channel dysfunction are conserved in distinct models of prodromal Parkinson’s disease. Sci. Rep..

[33] Ellens D.J., Leventhal D.K. (2013). Electrophysiology of Basal Ganglia and Cortex in Models of Parkinson Disease. J. Park.’s Dis..

[34] Lin M., Mckie P.M., Shaerzadeh F., Gamble-George J., Miller D.R., Martyniuk C.J., Khoshbouei H. (2021). In Parkinson’s patient-derived dopamine neurons, the triplication of α-synuclein locus induces distinctive firing pattern by impeding D2 receptor autoinhibition. Acta Neuropathol. Commun..

[35] Matuskey D., Tinaz S., Wilcox K.C., Naganawa M., Toyonaga T., Dias M., Henry S., Pittman B., Ropchan J., Nabulsi N. (2020). Synaptic Changes in Parkinson Disease Assessed with in vivo Imaging. Ann. Neurol..

[36] Saari L., Kivinen K., Gardberg M., Joutsa J., Noponen T., Kaasinen V. (2017). Dopamine transporter imaging does not predict the number of nigral neurons in Parkinson disease. Neurology.

[37] Creed R.B., Menallel L., Casey B., Dave K.D., Janssens H.B., Veinbergs I., van der Hart M., Rassoulpour A., Goldberg M.S. (2019). Basal and Evoked Neurotransmitter Levels in Parkin, DJ-1, PINK1 and LRRK2 Knockout Rat Striatum. Neuroscience.

[38] Chotibut T., Fields V., Salvatore M.F. (2014). Norepinephrine transporter inhibition with desipramine exacerbates L-DOPA-induced dyskinesia: Role for synaptic dopamine regulation in denervated nigrostriatal terminals. Mol. Pharmacol..

[39] Sarre S., Vandeneede D., Ebinger G., Michotte Y. (1990). Biotransformation of L-DOPA to dopamine in the substantia nigra of freely moving rats: Effect of dopamine receptor agonists and antagonists. J. Neurochem..

[40] Perez X.A., Parameswaran N., Huang L.Z., O’Leary K.T., Wuik M. (2008). Pre-synaptic dopaminergic compensation after moderate nigrostriatal damage in non-human primates. J. Neurochem..

[41] Mela F., Marti M., Bido S., Cenci M.A., Morari M. (2012). In vivo evidence for a differential contribution of striatal and nigral D1 and D2 receptors to l-DOPA induced dyskinesia and the accompanying surge of nigral amino acid levels. Neurobiol. Dis..

[42] Kliem M.A., Pare J.F., Khan Z.U., Wichmann T., Smith Y. (2010). Ultrastructural localization and function of dopamine D1-like receptors in the substantia nigra pars reticulata and the internal segment of the globus pallidus of parkinsonian monkeys. Eur. J. Neurosci..

[43] Mailman R.B., Yang Y., Huang X. (2021). D1, not D2, dopamine receptor activation dramatically improves MPTP-induced parkinsonism unresponsive to levodopa. Eur. J. Pharmacol..

[44] Trevitt J.T., Carlson B.B., Nowend K., Salamone J.D. (2001). Substantia nigra pars reticulate is a highly potent site of action for the behavioral effects of the D1 antagonist SCH23390 in rat. Psychopharmacology.

[45] Tang P., Knight W.C., Li H., Guo Y., Perlmutter J.S., Benzinger T.L.S., Morris J.C., Xu J. (2021). Dopamine D1 + D3 receptor density may correlate with parkinson disease clinical features. Ann. Clin. Transl. Neurol..

[46] Roedter A., Winkler C., Samil M., Walter G., Brandis A., Nikkhah G. (2001). Comparison of unilateral and bilateral intrastriatal 6-hydroxydopamine-induced axon terminal lesions: Evidence for interhemispheric functional coupling of the two nigrostriatal pathways. J. Comp. Neurol..

[47] Radnikow G., Misgeld U. (1998). Dopamine D_1_ receptors facilitate GABA_A_ synaptic currents in the rat substantia nigra pars reticulata. J. Neurosci..

[48] Dorval A.D., Grill W.M. (2014). Deep brain stimulation of the subthalamic nucleus reestablishes neuronal information transmission in the 6-OHDA rat model of parkinsonism. J. Neurophysiol..

[49] DeLong M.R., Wichmann T. (2015). Basal Ganglia Circuits as Targets for Neuromodulation in Parkinson Disease. JAMA Neurol..

[50] McGregor M.M., Nelson A.B. (2019). Circuit mechanisms of Parkinson’s disease. Neuron.

[51] Calabresi P., Picconi B., Tozzi A., Ghiglieri V., Di Filippo M. (2014). Direct and indirect pathways of basal ganglia: A critical reappraisal. Nat. Neurosci..

[52] Blesa J., Foffani G., Dehay B., Bezard E., Obeso J.A. (2022). Motor and non-motor circuit disturbances in early Parkinson disease: Which happens first? Nat. Rev. Neurosci..

[53] Zaman V., Boger H.A., Granholm A.C., Rohrer B., Moore A., Buhusi M., Gerhardt G.A., Hoffer B.J., Middaugh L.D. (2008). The nigrostriatal dopamine system of aging GFRalpha-1 heterozygous mice: Neurochemistry, morphology and behavior. Eur. J. Neurosci..

[54] Pruett B.S., Salvatore M.F. (2013). Nigral GFRα1 infusion in aged rats increases locomotor activity, nigral tyrosine hydroxylase, and dopamine content in synchronicity. Mol. Neurobiol..

[55] Gill S.S., Patel N.K., Hotton G.R., O’Sullivan K., McCarter R., Bunnage M., Brooks D.J., Svendsen C.N., Heywood P. (2003). Direct brain infusion of glial cell line-derived neurotrophic factor in Parkinson disease. Nat. Med..

[56] Grondin R., Cass W.A., Zhang Z., Stanford J.A., Gash D.M., Gerhardt G.A. (2003). Glial Cell Line-Derived Neurotrophic Factor Increases Stimulus-Evoked Dopamine Release and Motor Speed in Aged Rhesus Monkeys. J. Neurosci..

[57] Gash D.M., Zhang Z., Ovadia A., Cass W.A., Yi A., Simmerman L., Russell D., Martin D., Lapchak P.A., Collins F. (1996). Functional recovery in parkinsonian monkeys treated with GDNF. Nature.

[58] Gerhardt G.A., Cass W.A., Huettl P., Brock S., Zhang Z., Gash D.M. (1999). GDNF improves dopamine function in the substantia nigra but not the putamen of unilateral MPTP-lesioned rhesus monkeys. Brain Res..

[59] Hoffer B.J., Hoffman A.F., Bowenkamp K.E., Huettl P., Hudson J., Martin D., Lin L.F., Gerhardt G.A. (1994). Glial cell line-derived neurotrophic factor reverses toxin-induced injury to midbrain dopaminergic neurons in vivo. Neurosci. Lett..

[60] Lang A.E., Gill S.S., Patel N.K., Lozano A., Nutt J.G., Penn R., Brooks D.J., Hotton G., Moro E., Heywood P. (2006). Randomized controlled trial of intraputamenal glial cell line-derived neurotrophic factor infusion in Parkinson’s disease. Ann. Neurol..

[61] Salvatore M.F., Zhang J.L., Large D.M., Wilson P.E., Gash C.R., Thomas T.C., Haycock J.W., Bing G., Stanford J.A., Gash D.M. (2004). Striatal GDNF administration increases tyrosine hydroxylase phosphorylation in rat striatum and substantia nigra. J. Neurochem..

[62] Salvatore M.F., Gerhardt G.A., Dayton R.D., Klein R.L., Stanford J.A. (2009). Bilateral effects of unilateral GDNF administration on dopamine- and GABA-regulating proteins in the rat nigrostriatal system. Exp. Neurol..

[63] Kasanga E.A., Han Y., Navarrete W., McManus R., Shifflet M.K., Parry C., Barahona A., Manfredsson F.P., Nejtek V.A., Richardson J.R. (2023). Differential expression of RET and GDNF family receptor, GFR-α1, between striatum and substantia nigra following nigrostriatal lesion: A case for diminished GDNF-signaling. Exp. Neurol..

[64] Whone A., Luz M., Boca M., Woolley M., Mooney L., Dharia S., Broadfoot J., Cronin D., Schroers C., Barua N.U. (2019). Randomized trial of intermittent intraputamenal glial cell line-derived neurotrophic factor in Parkinson’s disease. Brain.

[65] Kordower J.H., Goetz C.G., Chu Y., Halliday G.M., Nicholson D.A., Musial T.F., Marmion D.J., Stoessl A.J., Freeman T.B., Olanow C.W. (2017). Robust graft survival and normalized dopaminergic innervation do not obligate recovery in a Parkinson disease patient. Ann. Neurol..

[66] Furukawa K., Shima A., Kambe D., Nishida A., Wada I., Sakamaki H., Yoshimura K., Terada Y., Sakato Y., Mitsuhashi M. (2022). Motor progression and nigrostriatal neurodegeneration in Parkinson’s disease. Ann. Neurol..

[67] Karimi M.K., Tian L., Flores H., Su Y., Tabbal S.D., Loftin S.K., Moerlin S.M., Perlmutter J.S. (2013). Validation of nigrostriatal positron emission tomography measures: Critical limits. Ann. Neurol..

[68] Perlmuttter J.S., Norris S.A. (2014). Neuroimaging biomarkers for Parkinson’s disease: Fact and fantasy. Ann. Neurol..

[69] Schröter N., Rijntjes M., Urbach H., Weiller C., Treppner M., Kellner E., Jost W.H., Sajonz B.E.A., Reisert M., Hosp J.A. (2022). 2022. Disentangling nigral and putaminal contribution to motor impairment and levodopa response in Parkinson’s disease. NPJ Park. Dis..

[70] Pérez-Taboada I., Alberquilla S., Martin E.D., Anand R., Vietti-Michelina S., Tebeka N.N., Cantley J., Cragg S.J., Moratalla R., Vallejo M. (2020). Diabetes Causes Dysfunctional Dopamine Neurotransmission Favoring Nigrostriatal Degeneration in Mice. Mov. Disord..

[71] Gonzalez-Rodriguez P., Zampese E., Stout K.A., Guzman J.N., Ilijic E., Yang B., Tkatch T., Stavarache M.A., Wokosin D.L., Gao L. (2021). Disruption of mitochondrial complex I induces progressive parkinsonism. Nature.

[72] Dave K.D., De Silva S., Sheth N.P., Ramboz S., Beck M.J., Quang C., Switzer R.C., Ahmad S.O., Sunkin S.M., Walker D. (2014). Phenotypic characterization of recessive gene knockout rat models of Parkinson’s disease. Neurobiol. Dis..

[73] Blesa J., Pifl C., Sánchez-González M.A., Juri C., García-Cabezas M.A., Adánez R., Iglesias E., Collantes M., Peñuelas I., Sánchez-Hernández J.J. (2012). The nigrostriatal system in the presymptomatic and symptomatic stages in the MPTP monkey model: A PET, histological, and biochemical study. Neurobiol. Dis..

[74] Petzinger G.M., Walsh J.P., Akopian G., Hogg E., Abernathy A., Arevalo P., Turnquist P., Vuckovic M., Fisher B.E., Togasaki D.M. (2007). Effects of treadmill exercise on dopaminergic transmission in the 1-methyl-4-phenyl-1,2,3,6-tetrahydropyridine-lesioned mouse model of basal ganglia injury. J. Neurosci..

[75] O’Dell S.J., Gross N.B., Fricks A.N., Casiano B.D., Nguyen T.B., Marshall J.F. (2007). Running wheel exercise enhances recovery from nigrostriatal dopamine injury without inducing neuroprotection. Neuroscience.

[76] Churchill M.J., Pflibsen L., Sconce M.D., Moore C., Kim K., Meshul C.K. (2017). Exercise in an animal model of Parkinson’s disease: Motor recovery but not restoration of the nigrostriatal pathway. Neuroscience.

[77] Robertson G.S., Robertson H.A. (1999). Evidence that L-DOPA-induced rotational behavior is dependent on both striatal and nigral mechanisms. J. Neurosci..

[78] Robertson G.S., Robertson H.A. (1988). Evidence that the substantia nigra is a site of action for L-DOPA. Neurosci. Lett..

[79] Jackson E.A., Kelly P.H. (1983). Role of nigral dopamine in amphetamine-induced locomotor activity. Brain Res..

[80] Bradbury A.J., Costall B., Kelly M.E., Naylor R.J., Smith J.A. (1985). Biochemical correlates of motor changes caused by the manipulation of dopamine function in the substantia nigra of the mouse. Neuropharmacology.

[81] Jackson E.A., Kelly P.H. (1984). Effects of intranigral injections of dopamine agonists and antagonists, glycine, muscimol and N-methyl-D,L-aspartate on locomotor activity. Brain Res. Bull..

[82] Ahlenius S., Anden N.E., Engel J. (1973). Restoration of locomotor activity in mice by low L-DOPA doses after suppression by alpha-methyltyrosine but not by reserpine. Brain Res..

[83] Dolphin A.C., Jenner P., Marsden C.D. (1976). The relative importance of dopamine and noradrenaline receptor stimulation for the restoration of motor activity in reserpine or alpha-methyl-p-tyrosine pre-treated mice. Pharmacol. Biochem. Behav..

[84] Salvatore M.F., Pruett B.S. (2012). Dichotomy of Tyrosine Hydroxylase and Dopamine Regulation between Somatodendritic and Terminal Field Areas of Nigrostriatal and Mesoaccumbens Pathways. PLoS ONE.

[85] Leng A., Mura A., Hengerer B., Feldon J., Ferger B. (2005). Effects of blocking the dopamine biosynthesis and of neurotoxic dopamine depletion with 1-methyl-4-phenyl-1,2,3,6-tetrahydropyridine (MPTP) on voluntary wheel running in mice. Behav. Brain Res..

[86] Paquette M.A., Marsh S.T., Hutchings J.E., Castañeda E. (2009). Amphetamine-evoked rotation requires newly synthesized dopamine at 14 days but not 1 day after intranigral 6-OHDA and is consistently dissociated from sensorimotor behavior. Behav. Brain Res..

[87] Ankenman R., Salvatore M.F. (2007). Low dose alpha-methyl-para-tyrosine (AMPT) in the treatment of dystonia and dyskinesia. J. Neuropsychiatry Clin. Neurosci..

[88] Bloemen O.J.N., de Koning M.B., Boot E., Booij J., van Amelsvoort T.A. (2008). Challenge and Therapeutic Studies Using Alpha-Methyl-para-Tyrosine (AMPT) in Neuropsychiatric Disorders: A Review. Cent. Nerv. Syst. Agents Med. Chem..

[89] Rubinstein M., Gershanik O., Stefano F.J. (1988). Different roles of D-1 and D-2 dopamine receptors involved in locomotor activity of supersensitive mice. Eur. J. Pharmacol..

[90] Lima A.C., Meurer Y.S.R., Bioni V.S., Cunha D.M.G., Goncalves N., Lopes-Silva L.B., Becegato M., Soares M.B.L., Marinho G.F., Santos J.R. (2021). Female Rats Are Resistant to Cognitive, Motor and Dopaminergic Deficits in the Reserpine-Induced Progressive Model of Parkinson’s Disease. Front. Aging Neurosci..

[91] Duty S., Jenner P. (2011). Animal models of Parkinson’s disease: A source of novel treatments and clues to the cause of the disease. Br. J. Pharmacol..

[92] May R.H., Voegele G.E. (1956). Parkinsonian reactions following chlorpromazine and reserpine; similar reactions in the same patients. AMA Arch. Neurol. Psychiatry.

[93] Nirenberg M.J., Chan J., Liu Y., Edwards R.H., Pickel V.M. (1996). Ultrastructural localization of the vesicular monoamine transporter-2 in midbrain dopaminergic neurons: Potential sites for somatodendritic storage and release of dopamine. J. Neurosci..

[94] Keller C.M., Salvatore M.F., Pruett B.S., Guerin G.F., Goeders N.E. (2011). Biphasic dopamine regulation in mesoaccumbens pathway in response to non-contingent binge and escalating methamphetamine regimens in the Wistar rat. Psychopharmacology.

[95] Nissbrandt H., Sundström E., Jonsson G., Hjorth S., Carolsson A. (1989). Synthesis and Release of Dopamine in Rat Brain: Comparison Between Substantia Nigra Pars Compacta, Pars Reticulata, and Striatum. J. Neurochem..

[96] Heeringa M.J., Abercrombie E.D. (1995). Biochemistry of Somatodendritic Dopamine Release in Substantia Nigra: An In Vivo Comparison with Striatal Dopamine Release. J. Neurochem..

[97] Santiago M., Westerink B.H.C. (1991). Characterization and Pharmacological Responsiveness of Dopamine Release Recorded by Microdialysis in the Substantia Nigra of Conscious Rats. J. Neurochem..

[98] Yee A.G., Forbes B., Cheung P.Y., Martini A., Burrell M.H., Freestone P.S., Lipski J. (2018). Action potential and calcium dependence of tonic somatodendritic dopamine release in the Substantia Nigra pars compacta. J. Neurochem..

[99] Cragg S.J., Rice M.E. (2004). Dancing past the DAT at a DA synapse. Trends Neurosci..

[100] Kaasinen V., Vahlberg T., Stoessl J.A., Strafella A.P., Antonini A. (2021). Dopamine receptors in Parkinson’s disease: A meta-analysis of imaging studies. Mov. Disord..

[101] Biswas B., Carlsson A. (1978). Potentiation by Neuroleptic Agents of the Inhibitory Action of Intraperitoneally Administered GABA on the Locomotor Activity of Mice. Pharmacol. Biochem. Behav..

[102] Hillegaart V., Ahlenius S. (1987). Effects of raclopride on exploratory locomotor activity, treadmill locomotion, conditioned avoidance behaviour and catalepsy in rats: Behavioural profile comparisons between raclopride, haloperidol and preclamol. Pharmacol. Toxicol..

[103] Löschmann P.A., Smith L.A., Lange K.W., Jaehnig P., Jenner P., Marsden C.D. (1991). Motor activity following the administration of selective D-1 and D-2 dopaminergic drugs to normal common marmosets. Psychopharmacology.

[104] Ericson H., Radesäter A.C., Servin E., Magnusson O., Mohringe B. (1996). Effects of intermittent and continuous subchronic administration of raclopride on motor activity, dopamine turnover and receptor occupancy in the rat. Pharmacol. Toxicol..

[105] Hoffman D.C., Beninger R.J. (1985). The D1 dopamine receptor antagonist, SCH 23390 reduces locomotor activity and rearing in rat. Pharmacol. Biochem. Behav..

[106] Schindler C.W., Caramona G.N. (2002). Effects of dopamine agonists and antagonists on locomotor activity in male and female rats. Pharmacol. Biochem. Behav..

[107] Svensson K.A., Heinz B.A., Schaus J.M., Beck J.P., Hao J., Krushinski J.H., Reinhard M.R., Cohen M.P., Hellman S.L., Getman B.G. (2017). An Allosteric Potentiator of the Dopamine D1 Receptor Increases Locomotor Activity in Human D1 Knock-In Mice without Causing Stereotypy or Tachyphylaxis. J. Pharmacol. Exp. Ther..

[108] Isaacson S.H., Hauser R.A., Pahwa R., Gray D., Duvvuri S. (2023). Dopamine agonists in Parkinson’s disease: Impact of D1-like or D2-like dopamine receptor subtype selectivity and avenues for future treatment. Clin. Park. Relat. Disord..

[109] Papapetropoulos S., Liu W., Duvvuri S., Thayer K., Gray D.L. (2018). Evaluation of D1/D5 partial agonist PF-06412562 in Parkinson’s disease following oral administration. Neurodegener. Dis..

[110] Huang X., Lewis M.M., Van Scoy L.J., De Jesus S., Eslinger P.J., Arnold A.C., Miller A.J., Fernandez-Mendoza J., Snyder B., Harrington W. (2020). The D1/D5 Dopamine Partial Agonist PF-06412562 in Advanced-Stage Parkinson’s Disease: A Feasibility Study. J. Park.’s Dis..

[111] Pothos E.N., Przedborski S., Davila V., Schmitz Y., Sulzer D. (1998). D_2_-Like Dopamine Autoreceptor Activation Reduces Quantal Size in PC12 Cells. J. Neurosci..

[112] Cragg S.J., Greenfield S.A. (1997). Differential Autoreceptor Control of Somatodendritic and Axon Terminal Dopamine Release in Substantia Nigra, Ventral Tegmental Area, and Striatum. J. Neurosci..

[113] Lanza K., Bishop C. (2021). Dopamine D3 Receptor Plasticity in Parkinson’s Disease and L-DOPA-Induced Dyskinesia. Biomedicines.

[114] Chagraoui A., Di Giovanni G., De Deurwaerdère P. (2022). Neurobiological and Pharmacological Perspectives of D3 Receptors in Parkinson’s Disease. Biomolecules.

[115] Castello J., Cortes M., Malave L., Kottmann A., Sibley D.R., Friedman E., Rebholz H. (2020). The Dopamine D5 receptor contributes to activation of cholinergic interneurons during L-DOPA induced dyskinesia. Sci. Rep..

[116] Chetrit J., Taupignon A., Froux L., Morin S., Bouali-Benazzouz R., Naudet F., Kadiri N., Gross C.E., Bioulac B., Benazzouz A. (2013). Inhibiting subthalamic D5 receptor constitutive activity alleviates abnormal electrical activity and reverses motor impairment in a rat model of Parkinson’s disease. J. Neurosci..

[117] Schultz W., Ruffieux A., Aebischer P. (1983). The activity of pars compacta neurons of the monkey substantia nigra in relation to motor activation. Exp. Brain Res..

[118] da Silva J.A., Tecuapetla F., Paixão V., Costa R.M. (2018). Dopamine neuron activity before action Initiation gates and invigorates future movements. Nature.

[119] Coddington L.T., Dudman J.T. (2019). Learning from Action: Reconsidering Movement Sinaling in Midbrain Dopamine Neuron Activity. Neuron.

[120] Klaus A., da Silva J.A., Costa R.M. (2019). What, If, and When to Move: Basal Ganglia Circuits and Self-Paced Action Initiation. Ann. Rev. Neurosci..

[121] Bergquist F., Shahabi H.N., Nissbrandt H. (2003). Somatodendritic dopamine release in rat substantia nigra influences motor performance on the accelerating rod. Brain Res..

[122] Hebert M.A., Gerhardt G.A. (1998). Normal and drug-induced locomotor behavior in aging: Comparison to evoked DA release and tissue content in Fischer 344 rats. Brain Res..

[123] Yurek D.M., Hipkens S.B., Hebert M.A., Gash D.M., Gerhardt G.A. (1998). Age-related decline in striatal dopamine release and motoric function in Brown Norway/Fischer 344 hybrid rats. Brain Res..

[124] Gaugler M.N., Genc O., Bobela W., Mohanna S., Ardah M.T., El-Agnaf O.M., Cantoni M., Bensadoun J.C., Schneggenburger R., Knott G.W. (2012). Nigrostriatal overabundance of α-synuclein leads to decreased vesicle density and deficits in dopamine release that correlate with reduced motor activity. Acta Neuropathol..

[125] Goodwin J.S., Larson G.A., Swant J., Sen N., Javitch J.A., Zahniser N.R., De Felice L.J., Khoshbouei H. (2009). Amphetamine and methamphetamine differentially affect dopamine transporters in vitro and in vivo. J. Biol. Chem..

[126] Kahlig K.M., Binda F., Khoshbouei H., Blakely R.D., McMahon D.G., Javitch J.A., Galli A. (2005). Amphetamine induces dopamine efflux through a dopamine transporter channel. Proc. Natl. Acad. Sci. USA.

[127] Sulzer D., Sonders M.S., Poulsen N.W., Galli A. (2005). Mechanisms of neurotransmitter release by amphetamines: A review. Prog. Neurobiol..

[128] Rivière G.J., Byrnes K.A., Gentry W.B., Owens S.M. (1999). Spontaneous Locomotor Activity and Pharmacokinetics of Intravenous Methamphetamine and Its Metabolite Amphetamine in the Rat. J. Pharmacol. Exp. Ther..

[129] Laruelle M., Abi-Dargham A., van Dyck C.H., Rosenblatt W., Zea-Ponce Y., Zoghbi S.S., Baldwin R.M., Charney D.S., Hoffer P.B., Kung H.F. (1995). SPECT Imaging of Striatal Dopamine Release after Amphetamine Challenge. J. Nuclear Med..

[130] Hall D.A., Stanis J.J., Avila H.M., Gulley J.M. (2008). A comparison of amphetamine- and methamphetamine-induced locomotor activity in rats: Evidence for qualitative differences in behavior. Psychopharmacology.

[131] Ciliax B.J., Heilman C., Demchyshyn L.L., Pristupa Z.B., Ince E., Hersch S.M., Niznik H.B., Levey A.I. (1995). The dopamine transporter: Immunochemical characterization and localization in brain. J. Neurosci..

[132] Nirenberg M.J., Vaughan R.A., Uhl G.R., Kuhar M.J., Pickel V.M. (1996). The Dopamine Transporter Is Localized to Dendritic and Axonal Plasma Membranes of Nigrostriatal Dopaminergic Neurons. J. Neurosci..

[133] Vaughan R.A., Foster J.D. (2013). Mechanisms of dopamine transporter regulation in normal and disease states. Trends Pharmacol. Sci..

[134] Hoffman A.F., Lupica C.R., Gerhardt G.A. (1998). Dopamine transporter activity in the substantia nigra and striatum assessed by high-speed chronoamperometric recordings in brain slices. J. Pharmacol. Exp. Ther..

[135] Ford C.P., Gantz S.C., Phillips P.E.M., Williams J.T. (2010). Control of extracellular dopamine at dendrite and axon terminals. J. Neurosci..

[136] Cragg S.J., Rice M.E., Greenfield S.A. (1997). Heterogeneity of Electrically Evoked Dopamine Release and Reuptake in Substantia Nigra, Ventral Tegmental Area, and Striatum. J. Neurophysiol..

[137] Ma S.Y., Ciliax B.J., Stebbins G., Jaffar S., Joyvce J.N., Cochran E.J., Kordower J.H., Mash D.C., Levey A.I., Mufson E.J. (1999). Dopamine transporter-immunoreactive neurons decrease with age in the human substantia nigra. J. Comp. Neurol..

[138] Salvatore M.F., Apparsundaram S., Gerhardt G.A. (2003). Decreased plasma membrane expression of striatal dopamine transporter in aging. Neurobiol. Aging.

[139] Bu M., Farrer M.J., Khoshbouei H. (2021). Dynamic control of the dopamine transporter in neurotransmission and homeostasis. NPJ Park. Dis..

[140] Jones D.R., Gainetdinov R.R., Jaber M., Giros B., Wightman R.M., Caron M.G. (1998). Profound neuronal plasticity in response to inactivation of the dopamine transporter. Proc. Natl. Acad. Sci. USA.

[141] Salvatore M.F., Calipari E.S., Jones S.R. (2016). Regulation of tyrosine hydroxylase expression and phosphorylation in dopamine transporter-deficient mice. ACS Chem. Neurosci..

[142] Morón J.A., Brockiington A., Wise R.A., Rocha B.A., Hope B.T. (2002). Dopamine Uptake through the Norepinephrine Transporter in Brain Regions with Low Levels of the Dopamine Transporter: Evidence from Knock-Out Mouse Lines. J. Neurosci..

[143] Chotibut T., Apple D.M., Jefferis R., Salvatore M.F. (2012). Dopamine Transporter Loss in 6-OHDA Parkinson’s Model Is Unmet by Parallel Reduction in Dopamine Uptake. PLoS ONE.

[144] Reinwald J.R., Gass N., Mallien A.S., Sartorius A., Becker R., Sack M., Falfan-Melgoza C., von Hohenberg C.C., Leo D., Pfeiffer N. (2022). Dopamine transporter silencing in the rat: Systems-level alterations in striato-cerebellar and prefrontal-midbrain circuits. Mol. Psychiatry.

[145] Giros B., Jaber M., Jones S.R., Wightman R.M., Caron M.G. (1996). Hyperlocomotion and indifference to cocaine and amphetamine in mice lacking the dopamine transporter. Nature.

[146] Spielewoy C., Roubert C., Hamon M., Nosten-Bertrand M., Betancur C., Giros B. (2000). Behavioural disturbances associated with hyperdopaminergia in dopamine-transporter knockout mice. Behav. Pharmacol..

[147] Stanford J.A., Vorontsova E., Surgener S.P., Gerhardt G.A., Fowler S.C. (2002). Aged Fischer 344 rats exhibit altered locomotion in the absence of decreased locomotor activity: Exacerbation by nomifensine. Neurosci. Lett..

[148] Salvatore M.F., Kasanga E.A., Kelly D.P., Venable K.E., McInnis T.R., Cantu M.A., Terrebonne J., Lanza K., Meadows S.M., Centner A. (2023). Modulation of nigral dopamine signaling mitigates parkinsonian signs of aging: Evidence from intervention with caloric restriction or inhibition of dopamine uptake. GeroScience.

[149] Salvatore M.F., Terrebonne J., Cantu M.A., McInnis T., Venable K., Kelley P., Kasanga E.A., Latimer B., Owens C.L., Pruett B.S. (2017). Dissociation of striatal dopamine and tyrosine hydroxylase expression from aging-related motor decline: Evidence from calorie restriction intervention. J. Gerontol. A Biol. Sci. Med. Sci..

[150] Salvatore M.F., Terrebonne J., Fields V., Nodurft D., Runfalo C., Latimer B., Ingram D.K. (2016). Initiation of calorie restriction in middle-aged male rats attenuates aging-related motoric decline and bradykinesia without increased striatal dopamine. Neurobiol. Aging.

[151] Salvatore M.F., Pruett B.S., Spann S.L., Dempsey C. (2009). Aging Reveals a Role for Nigral Tyrosine Hydroxylase ser31 Phosphorylation in Locomotor Activity Generation. PLoS ONE.

[152] Robertson G., Damsma G., Fibiger H. (1991). Characterization of dopamine release in the substantia nigra by in vivo microdialysis in freely moving rats. J. Neurosci..

[153] Nieoullon A., Cheramy A., Glowinski J. (1977). Nigral and striatal dopamine release under sensory stimuli. Nature.

[154] Coddington L.T., Dudman J.T. (2018). The timing of action determines reward prediction signals in identified midbrain dopamine neurons. Nat. Neurosci..

[155] Salvatore M.F., McInnnis T.R., Cantu M.A., Apple D.M., Pruett B.S. (2019). Tyrosine Hydroxylase Inhibition in Substantia Nigra Decreases Movement Frequency. Mol. Neurobiol..

[156] Andersson D.R., Nissbrandt H., Bergquist F. (2006). Partial depletion of dopamine in substantia nigra impairs motor performance without altering striatal dopamine neurotransmission. Eur. J. Neurosci..

[157] Salvatore M.F., Garcia-Espana A., Goldstein M., Deutch A.Y., Haycock J.W. (2000). Stoichiometry of tyrosine hydroxylase phosphorylation in the nigrostriatal and mesolimbic systems in vivo: Effects of acute haloperidol and related compounds. J. Neurochem..

[158] Salvatore M.F., Pruett B.S., Dempsey C., Fields V. (2012). Comprehensive profiling of dopamine regulation in substantia nigra and ventral tegmental area. J. Vis. Exp..

[159] Fearnley J.M., Lees A.J. (1991). Ageing and Parkinson’s disease: Substantia nigra regional selectivity. Brain.

[160] Emborg M.E., Ma S.Y., Mufson E.J., Levey A.I., Taylor M.D., Brown W.D., Holden J.E., Kordower J.H. (1998). Age-related declines in nigral neuronal function correlate with motor impairments in rhesus monkeys. J. Comp. Neurol..

[161] Chu Y., Hirst W.D., Federoff H.J., Harms A.S., Stoessl A.J., Kordower J.H. (2023). Nigrostriatal tau pathology in parkinsonism and Parkinson’s disease. Brain.

[162] Ross G.W., Petrovich H., Abbott R.D., Nelson J., Markesbery W., Davis D., Hardman J., Launer L., Masaki K., Tanner C.M. (2004). Parkinsonian signs and substantia nigra neuron density in descendent elders without PD. Ann. Neurol..

[163] Buchman A.S., Shulman J.M., Nag S., Leurgans S.E., Arnold S.E., Morris M.C., Schneider J.A., Bennett D.A. (2012). Nigral pathology and parkinsonian signs in elders without Parkinson disease. Ann. Neurol..

[164] Gerhardt G.A., Cass W.A., Yi A., Zhang Z., Gash D.M. (2002). Changes in somatodendritic but not terminal dopamine regulation in aged rhesus monkeys. J. Neurochem..

[165] Irwin I., DeLanney L.E., McNeill T., Chan P., Forno L.S., Murphy G.M., Di Monte D.A., Sandy M.S., Langston J.W. (1994). Aging and the nigrostriatal dopamine system: A non-human primate study. Neurodegeneration.

[166] Siddiqi Z., Kemper T.L., Killiany R. (1999). Age-related Neuronal Loss from the Substantia Nigra-Pars Compacta and Ventral Tegmental Area of the Rhesus Monkey. J. Neuropathol. Exp. Neurol..

[167] Wolf M.E., LeWitt P.A., Bannon M.J., Dragovic L.J., Kapatos G. (1991). Effect of aging on tyrosine hydroxylase protein content and the relative number of dopamine nerve terminals in human caudate. J. Neurochem..

[168] Kish S.J., Shannak K., Rajput A., Deck J.H.N., Hornykiewicz O. (1992). Aging produces a specific pattern of striatal dopamine loss: Implications for the etiology of idiopathic Parkinson’s disease. J. Neurochem..

[169] Haycock J.W., Becker L., Ang L., Yoshiaki F., Hornykiewicz O., Kish S.J. (2003). Marked disparity between age-related changes in dopamine and other presynaptic dopaminergic markers in human striatum. J. Neurochem..

[170] Bernheimer H., Birkmayer W., Hornykiewicz O., Jellinger K., Seitelberger F. (1973). Brain dopamine and the syndromes of Parkinson and Huntington. Clinical, morphological and neurochemical correlations. J. Neurol. Sci..

[171] Marsden C.D. (1990). Parkinson’s disease. Lancet.

[172] Collier T.J., Lipton J., Daley B.F., Palfi S., Chu Y., Sortwell C., Bakay R.A., Sladek J.R., Kordower J.H. (2007). Aging-related changes in the nigrostriatal dopamine system and the response to MPTP in nonhuman primates: Diminished compensatory mechanisms as a prelude to parkinsonism. Neurobiol. Dis..

[173] Pifl C., Hornykiewicz O. (2006). Dopamine turnover is upregulated in the caudate/putamen of asymptomatic MPTP-treated rhesus monkeys. Neurochem. Int..

[174] Zigmond M.J. (1997). Do compensatory processes underlie the preclinical phase of neurodegenerative disease? Insights from an animal model of parkinsonism. Neurobiol. Dis..

[175] Blesa J., Trigo-Damas I., Dileone M., Lopez-Gonzalez del Rey N., Hernandez L.F., Obeso J.A. (2017). Compensatory mechanisms in Parkinson’s disease: Circuits adaptations and role in disease modification. Exp. Neurol..

[176] Sarre S., Yuan H., Jonkers N., Van Hemelrijck A., Ebinger G., Michotte Y. (2004). In vivo characterization of somatodendritic dopamine release in the substantia nigra of 6-hydroxydopamine-lesioned rats. J. Neurochem..

[177] Suhara T., Fukuda H., Inoue O., Itoh T., Suzuki K., Yamasaki T., Tateno Y. (1991). Age-related changes in human D1 dopamine receptors measured by positron emission tomography. Psychopharmacology.

[178] Hormigo S., Zhou J., Castro-Alamancos M.A. (2020). 2020. Zona incerta GABAergic output controls a signaling locomotor action in the midbrain tegmentum. eNeuro.

[179] Bezard E., Gray D., Kozak R., Leoni M., Cari C., Duvvuri S. (2024). Rationale and Development of Tavapadon, a D1/D5-Selective Partial Dopamine Agonist for the Treatment of Parkinson’s Disease. CNS Neurol. Disord. Drug Targets.

[180] Shui H.A., Peng Y.I., Wu R.M., Tsai Y.F. (2000). Evaluation of l-DOPA biotransformation during repeated l-DOPA infusion into the striatum in freely-moving young and old rats. Dev. Brain Res..

[181] Orosz D., Bennett J.P. (1992). Simultaneous microdialysis in striatum and substantia nigra suggests that the nigra is a major site of action of l-dihydroxyphenylalanine in the “Hemiparkinsonian” rat. Exp. Neurol..

[182] Sarre S., Herregodts P., Deleu D., Devrieze A., De Klippel N., Ebinger G., Michotte Y. (1992). Biotransformation of L-DOPA in striatum and substantia nigra of rats with a unilateral, nigrostriatal lesion: A microdialysis study. Naunyn-Schmiedeberg’s Arch. Pharmacol..

[183] Grondin R., Zhang Z., Yi A., Cass W.A., Maswood N., Andersen A.H., Elsberry D.D., Klein M.C., Gerhardt G.A., Gash D.M. (2002). Chronic, controlled GDNF infusion promotes structural and functional recovery in advanced parkinsonian monkeys. Brain.

[184] Slevin J.T., Gash D.M., Smith C.D., Gerhardt G.A., Kryscio R., Chebrolu H., Walton A., Wagner R., Young A.B. (2012). Unilateral intraputamenal glial cell line-derived neurotrophic factor in patients with Parkinson disease: Response to 1 year of treatment and 1 year of withdrawal. J. Neurosurg..

[185] Patel N.K., Pavese N., Javed S., Hotton G.R., Brooks D.J., Gill S.S. (2013). Benefits of putaminal GDNF infusion in Parkinson disease are maintained after GDNF cessation. Neurology.

[186] Slevin J.T., Gerhardt G.A., Smith C.D., Gash D.M., Kryscio R., Young B. (2005). Improvement of bilateral motor functions in patients with Parkinson disease through the unilateral intraputamenal infusion of glial cell line-derived neurotrophic factor. J. Neurosurg..

[187] Kasanga E.A., Owens C.L., Cantu M.A., Richard A.D., Davis R.W., McDivitt L.M., Blancher B., Pruett B.S., Tan C., Gajewski A. (2019). GFR-α1 Expression in Substantia Nigra Increases Bilaterally Following Unilateral Striatal GDNF in Aged Rats and Attenuates Nigral Tyrosine Hydroxylase Loss Following 6-OHDA Nigrostriatal Lesion. ACS Chem. Neurosci..

[188] Barker R.A., Bjorklund A., Gash D.M., Whone A., Van Laar A., Kordower J.H., Bankiewicz K., Kieburtz K., Saarma M., Booms S. (2020). GDNF and Parkinson’s Disease: Where Next? A Summary from a Recent Workshop. J. Park. Dis..

[189] Tomac A., Widenfalk J., Lin L.F. (1995). Retrograde axonal transport of glial cell line-derived neurotrophic factor in the adult nigrostriatal system suggests a trophic role in the adult. Proc. Natl. Acad. Sci. USA.

[190] Ibáñez C.F., Andressoo J.O. (2017). Biology of GDNF and its receptors—Relevance for disorders of the central nervous system. Neurobiol. Dis..

[191] Leitner M.L., Molliver D.C., Osborne P.A., Vejsada R., Golden J.P., Lampe P.A., Kato A.C., Milbrandt J., Johnson E.M. (1999). Analysis of the retrograde transport of glial cell line-derived neurotrophic factor (GDNF), Neurturin, and Persephin suggests that in vivo signaling for the GDNF family is GFRα coreceptor-specific. J. Neurosci..

[192] Salvatore M.F., Ai Y., Fischer B., Zhang A.M., Grondin R.C., Zhang Z., Gerhardt G.A., Gash D.M. (2006). Point source concentration of GDNF may explain failure of phase II clinical trial. Exp. Neurol..

[193] Geffen L.B., Jessell T.M., Cuello A.C., Iversen L.L. (1976). Release of dopamine from dendrites in rat substantia nigra. Nature.

[194] Cheramy A., Leviel V., Glowinski J. (1981). Dendritic release of dopamine in the substantia nigra. Nature.

[195] Waszczak B.L., Walters J.R. (1983). Dopamine modulation of the effects of gamma-aminobutyric acid on substantia nigra pars reticulata neurons. Science.

[196] Ruffieux A., Schultz W. (1980). Dopaminergic activation of reticulate neurons in the substantia nigra. Nature.

[197] 198 Matuszewich L., Yamamoto B.K. (1999). Modulation of GABA release by dopamine in the substantia nigra. Synapse.

[198] Lahiri A.K., Bevan M.D. (2020). Dopaminergic transmission rapidly and persistently enhances excitability of D1 receptor-expressing striatal projection neurons. Neuron.

[199] Collier T.J., Kanaan N.M., Kordower J.H. (2017). Aging and Parkinson’s disease: Different sides of the same coin?. Mov. Disord..

[200] Nejtek V.A., James R.N., Salvatore M.F., Alphonso H.M., Boehm G.W. (2021). Premature cognitive decline in specific domains found in young veterans with mTBI coincide with elder normative scores and advanced-age subjects with early-stage Parkinson’s disease. PLoS ONE.

[201] Berg D., Borghammer P., Fereshtehnejad S.M., Heinzel S., Horsager J., Schaeffer E., Psotuma R.B. (2021). Prodromal Parkinson disease subtypes-key to understanding heterogeneity. Nat. Rev. Neurosci..

[202] Salvatore M.F., Soto I., Alphonso H., Cunningham R., Nejtek V.A. (2021). Is there a neurobiological rationale for the utility of the Iowa Gambling Task in Parkinson’s disease?. J. Park. Dis..

